# The Cosmetic Results of a Simple Method for Repairing Preputial Skin Defect in Hypospadias

**Published:** 2014-07-04

**Authors:** Maryam Ghavami-Adel, Mansour Mollaeean, Nakysa Hooman

**Affiliations:** 1 Department of Pediatric Surgery, Aliasghar Pediatric Hospital, Iran University of Medical Science; 2 Department of Surgery, Valiasr Hospital, Tehran University of Medical Sciences; 3 Department of Pediatric Nephrology, Aliasghar Pediatric Hospital, Iran University of Medical Sciences, Tehran, Iran

**Keywords:** Hypospadias, Urethroplasty, Cosmetic Surgery, Penis

## Abstract

***Objective:*** Hypospadias is a common birth defect of the penis. Besides the abnormal position of the urethral opening, there is usually a ventral preputial defect with preputial redundancy in dorsal shaft. There are many flap procedures for correcting this defect. Here, we present our experience of skin coverage procedure with better cosmetic results.

***Methods:*** It is a prospective study on patients with mid-shaft to glandular hypospadias operated from June 2008 to December 2012. The operations were performed by one surgeon in two hospitals and the cosmetic results were evaluated by the surgeon, parents, and another pediatric surgeon by a satisfaction questionnaire. In this procedure, inner prepuce was incised curvilinearly, remaining 5 mm in medial and 8 mm in lateral aspects of the inner prepuce. For skin repair, dorsal flaps were approximated in midline along median raphe.

***Findings:*** Sixty-three patients with mean age of 25.75±8.46 (7-93) months were followed up for 7.06±3.34 (2-15( months. There were 4 complications. The overall satisfaction with penile skin coverage was 93.7% for parents and 98.4% for surgeons. Patients’ age and primary site of meatus had a significant correlation with cosmetic results (*P*<0.05), while urethroplasty techniques and post-operative complications were not significant.

***Conclusion:*** Reapproximation of dorsal flaps in midline is a simple method and can be used in most cases of uncomplicated primary hypospadias. By this technique a more normal appearance can be achieved.

## Introduction

Hypospadias is one of the most common congenital anomalies of the genito-urinary tract with an incidence rate of approximately 3.8 per 1000 male births^[^^1^^-^^3^^]^. Various operative techniques have been developed through the past decades^[^^4^^]^. They aim to achieve a more cosmetically normal penile appearance, straight urinary flow from a meatus with a normal position and straight erect penis^[^^3^^]^. 

 In addition to the abnormal position of the urethral opening, there is generally a ventral preputial deficiency with preputial redundancy in dorsal shaft that influences the appearance of the penis. 

 Regarding the perception of body image, evaluated on the validated Junior Genital Perception Scale (JGPS), hypospadias patients are more dissatisfied with their body image rather than controls^[^^4^^]^. Also the quality of life of kids with hypospadias might be deteriorated by negative penile self-perception, feeling of shame, or tantalizing remarks from their counterparts^[^^5^^]^. Additionally, an unsatisfactory cosmetic result can also lead to poorer school performance^[^^6^^]^

 To improve cosmetic appearance in hypospadias patients, different cosmetic surgical techniques have been introduced, but it is accepted that there is no particular technique appropriate or useful for all patients. As in previously described methods final appearance of penile skin coverage was not satisfactory and some of them are time consuming and very delicate. Here, we describe cosmetic results of a simple technique for skin coverage, which is suitable for repairing preputial skin defect in hypospadias.

## Subjects and Methods

This is a prospective study performed on patients with mid-shaft to glandular hypospadias operated from June 2008 to December 2012. All the operations were performed by the same pediatric surgeon in two centers (Aliasqar Pediatric Hospital,Tehran, and Alborz Social Security Hospital, Karaj, Iran). Undergoing preputial skin defect repair with our modified method (explained below), the cosmetic results were evaluated by parents, the surgeon, and another pediatric surgeon according to a scoring scale from 0 to 2 (0: no satisfaction, 1: not completely satisfied, 2: satisfied). We used this three points questionnaire according to PPPS (Pediatric Penile Perception Score)^[^^1^^,^^3^^,^^13^^,17]^. Although the final outcome of a good hypospadias repair evaluated by micturation, uroflowmetry, cosmesis, sexuality and relationship^ [^^3^^]^, according to age and accessibility of the patients we just evaluated the cosmesis. But for simplicity in use especially for parents we made some changes. The patients were followed up for 1 week, 3 months, and 6 months, and in some cases longer, according to the parents' demand after surgery. The collected data included the satisfaction level of cosmetic appearance, type of hypospadias, presence of chordee, age at the time of surgery, type of primary repair technique for urethra and foreskin, complications of surgery (bleeding, wound infection, skin necrosis or sloughing). All preputial skin defect repairs performed using the surgical technique described below were included in the study, but those performed using any other technique were excluded. The questionnaires were filled by surgeons and parents three times and internal consistency, validity and reliability determined by Cronbach’s alpha. Cronbach’s alpha of more than 0.07 was acceptable. We used 2*2 K square tables to compare frequency. *P*<0.05 was significant.

**Fig. 1:**
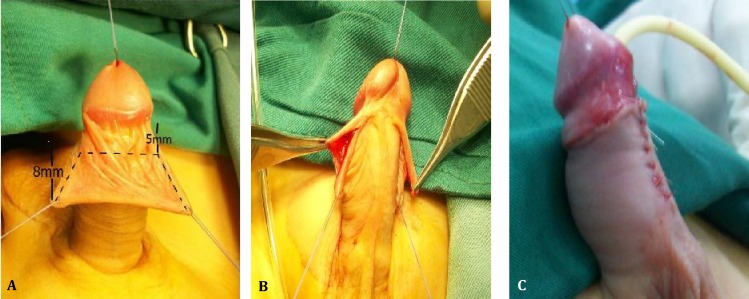
**1A.** Dotted lines indicate primary incision lines.** 1B**. Ventral aspect. 8mm length of remaining inner prepuce. **1C.** Approximation of inner prepuce and prepuce in midline in continuity of the median raphe.

*Surgical technique:* In this simple procedure, after inserting stay sutures at the edge of preputial margins, inner prepuce was incised curvilinear, remaining 5 mm in dorsal and 8 mm in ventral aspects of the inner prepuce (Fig 1A, 1B). In this way, we could achieve3 mm more of preputial defect in ventral side. After urethroplasty and glanduloplasty, with either MAGPI or TIP based on case, the inner prepuce was approximated to ventrum, and then the preputial skin divided in dorsum and reapproximated along with inner prepuce in the midline in continuity of median raphe (Fig.1C). The excess of prepuce was excised in laterals and the repair was completed with suturing to the inner prepuce. The preputial layers of foreskin were repaired with separate 6-0 polyglactin (VICRYL). Dressings were like other kinds of repair. Cephazolin was used as prophylactic antibiotic, also administered two doses after surgery. Acetaminophen syrup in combination with opioids was used as analgesic. All patients were invited to post operative follow-up. 

 Ethics: We obtained the informed consent of all subjects in this study, through their parents. It was also evaluated with pediatric surgeon internal committee and accepted by hospital surgery group committee.

## Findings

This study included 63 patients operated from June 2008 to December 2012. Patients’ age ranged from 7 months to 7 years (mean 25.75±8.46 months) with the mean follow up time of 7.06±3.34 months (2-15 months).

 The techniques of urethroplasty were MAGPI (36 cases) and TIP (27 cases). The modified method of preputial skin defect repair was employed in 54 patients operated. This technique was not employed in 9 boys either because there was no need for excess skin coverage in ventrum (n=3) or because of severe skin defect or deformity of the prepuce (hooked ones) (n=6). For these cases, we used other kinds of flaps (Byars flap n=1, Buttoner technique n=1, Onlay flap n=4). 

 Meatal positions were glandular in 16 patients (25.39%), coronal in 22 patients (34.91%), distal shaft in 17 patients (26.98%), and mid shaft in 8 patients (12.69%). 

 Follow ups showed that among 54 patients, only in 2 (3.70%) cases there were complications not related to the technique (2 urinary retentions). There was no skin necrosis. Overall satisfaction with this method was 93.7% for parents and 98.4% for surgeons. Patient age and primary site of meatus had a significant correlation with cosmetic results (*P*=0.002 and 0.01 respectively), as younger patients had better cosmetic results than older patients. Urethroplasty techniques and post-operative complications had little effect on cosmetic results. (*P*=0.8 and 0.1 respectively). There was a significant correlation between chordae and cosmetic results (*P*<0.001); chordae cause poorer cosmetic results.

## Discussion

After orthoplasty and urethroplasty, the penis must be covered with skin. Although numerous techniques have been developed for this purpose, many of them have been nowadays neglected^[^^10^^]^

 This study showed there is a high degree of satisfaction with this simple method of preputial skin repair.

 Acceptable external genital appearance is very important as it may have a great influence on normal psychosexual development^[^^4^^]^. In most studies self reported questionnaire (PPPS) about cosmetic satisfaction is an acceptable measure^[^^3^^,^^9^^-^^11^^]^. As the aim of our study was to evaluate the cosmetic results after a specific technique for ventral defect coverage we just used the forth component of the PPPS. In another study^[^^12^^]^ it was mentioned that in distal hypospadias, subjective evaluation of gross penile appearance can be a reliable measurement. As our patients at the time of study were children we took their parents’ idea.

 The technique described here is very simple and does not need much additional effort at the ending of an extensive operation. It seems that our technique is very similar to Snodgrass et al recommendation for alternating Byars’ flaps^[^^13^^]^ and that of Abou Zeid^[^^14^^]^, but there is a small modification. Since in our primary incision we follow sleeve resection rules for circumcision (5 mm in dorsal and 8 mm in ventral) we find a more normal appearing penis. There is also 3 mm more from the inner prepuce in ventral aspect that can help to cover ventral defect. As we employ most of the excess of the foreskin, there is no redundancy in ventrum. And as the suture line is in midline, penis has more normal appearance. 

 Cosmetic result of this method was very fine and we can prevent from penile rotation, which had been observed in up to 60% of patients undergoing hypospadias repair with tabularized preputial flaps (Leveuf technique)^[^^15^^]^. Necrosis is also one of the complications of methods that use flap for skin repairs. In this method, necrosis was not observed in any cases of skin repair because tissue blood flow was not disturbed.

 Our results showed there is a significant correlation between primary site of meatus and cosmetic results while mid-shaft position of meatus yielded less satisfaction compared with other kinds of hypospadias which may be because of chordae. As we reported in results session, chordae cause poorer cosmetic results. All cases that had mid-shaft position of meatus had chordae that may influence cosmetic results of hypospadias surgery. 

 Patient age had also significant correlation with cosmetic results, as satisfaction with cosmetic results was higher in younger patients. We suppose it may be due to the smaller size of penis, so they have fewer defects therefore they need less material for repair.

 As the incidence of complications after surgery was low (n=2) and the complications were due to principle surgery (not preputial skin defect repair) we did not consider the correlation between complications and hypospadias surgery. 

 In hypospadias repair values other than shaft skin appearance, such as meatus and glans, have influence on cosmetic outcome that we did not evaluate them in our study. On the other hand, according to Zaontz^[^^11^^] ^”Here is a classic study” that clearly shows that “beauty is in the eyes of the beholder, which in this instance is the patient himself.” Because of short duration of our study and age of the patients we just evaluated the parents’ idea not that of the patients themselves.

## Conclusion

Proper inner prepuce incision and reapproxi-mation of dorsal flaps in midline is a simple method and can be used in most cases of uncomplicated primary proximal hypospadias. This method has some advantages including: more near normal appearance and better cosmetic results, absence of necrosis and vascular compromise. As previously mentioned here we just evaluated parents and surgeons’ idea because of the young age of the patients. It seems longer follow up and serial evaluation of the patients to be appropriate.
